# Kappa Free Light Chains in the Context of Blood Contamination, and Other IgA- and IgM-Related Cerebrospinal Fluid Disease Pattern

**DOI:** 10.3390/cells10030616

**Published:** 2021-03-11

**Authors:** Malte Johannes Hannich, Alexander Dressel, Kathrin Budde, Astrid Petersmann, Matthias Nauck, Marie Süße

**Affiliations:** 1 Institute of Clinical Chemistry and Laboratory Medicine, University Medicine Greifswald, 17475 Greifswald, Germany; kathrin.budde@med.uni-greifswald.de (K.B.); astrid.petersmann@uni-oldenburg.de (A.P.); matthias.nauck@med.uni-greifswald.de (M.N.); 2 Department of Neurology, Carl-Thiem Hospital, 03048 Cottbus, Germany; A.Dressel@ctk.de; 3 Institute for Clinical Chemistry and Laboratory Medicine, University Oldenburg, 26129 Oldenburg, Germany; 4 DZHK (German Centre for Cardiovascular Research), Partner Site Greifswald, University Medicine, 17475 Greifswald, Germany; 5 Department of Neurology, University Medicine Greifswald, 17475 Greifswald, Germany; marie.suesse@med.uni-greifswald.de

**Keywords:** cerebrospinal fluid, free light chain kappa, immunoglobulin, artificial blood contamination, protein analytic, CSF

## Abstract

In this retrospective, monocentric cohort study, we tested if an intrathecal free light chain kappa (FLC-k) synthesis reflects not only an IgG but also IgA and IgM synthesis. We also analysed if FLC-k can help to distinguish between an inflammatory process and a blood contamination of cerebrospinal fluid (CSF). A total of 296 patient samples were identified and acquired from patients of the department of Neurology, University Medicine Greifswald (Germany). FLC-k were analysed in paired CSF and serum samples using the Siemens FLC-k kit. To determine an intrathecal FLC-k and immunoglobulin (Ig) A/-M-synthesis we analysed CSF/serum quotients in quotient diagrams, according to Reiber et al. Patient samples were grouped into three cohorts: cohort I (*n* = 41), intrathecal IgA and/or IgM synthesis; cohort II (*n* = 16), artificial blood contamination; and the control group (*n* = 239), no intrathecal immunoglobulin synthesis. None of the samples had intrathecal IgG synthesis, as evaluated with quotient diagrams or oligoclonal band analysis. In cohort I, 98% of patient samples presented an intrathecal synthesis of FLC-k. In cohort II, all patients lacked intrathecal FLC-k synthesis. In the control group, 6.5% presented an intrathecal synthesis of FLC-k. The data support the concept that an intrathecal FLC-k synthesis is independent of the antibody class produced. In patients with an artificial intrathecal Ig synthesis due to blood contamination, FLC-k synthesis is lacking. Thus, additional determination of FLC-k in quotient diagrams helps to discriminate an inflammatory process from a blood contamination of CSF.

## 1. Introduction

The detection of intrathecal immunoglobulin (Ig) synthesis as evidence of central nervous system (CNS) inflammation is an important aspect of cerebrospinal fluid (CSF) analysis. One method to distinguish blood derived from intrathecal, synthesized IgG, -A, or -M is the interpretation of IgG, -A, -M CSF and serum quotients (QIgG, QIgA, and QIgM; *y*-axis) in reference to the albumin CSF, and the serum quotient (Q-alb; *x*-axis) in relation to the empirically-derived hyperbolic reference range established by Reiber et al. [1]. This concept incorporates the CSF flow rate as the major influence on CSF protein concentration. Since albumin is exclusively synthesized extrathecally, the level of the albumin quotient reflects the integrity of the blood–CSF barrier function. The passage from blood into CSF of an extrathecally synthesized protein is also dependent on the molecular size of this protein, which is why the concentration of IgM in CSF is very low, followed by the concentrations of IgA and IgG [1]. However, for the detection of synthesis of intrathecal IgG, but not IgA or -M, the most sensitive method is the analysis of oligoclonal bands (OCB) by isoeletric focusing and immunodetection [2,3].

Kappa free light chains (FLC-k) have recently gained new interest as a similar sensitive method to detect intrathecal inflammation compared to OCB detection [4]. Some authors, however, point to the lesser specificity of FLC-k compared to OCB, as many patients with missing OCB still present higher FLC-k values [5]. To date, there is no consensus regarding the significance of these FLC-k values in patients with or without an inflammatory CNS disease.

FLC-k is synthesized in excess during the generation and assembly of Ig [6,7,8]. Consequently, it could not only represent intrathecal IgG synthesis, but also intrathecal IgA or IgM synthesis. This is of importance, as many diseases induce an immunoglobulin pattern with a dominance in IgM (e.g., neuroborreliosis, mumps meningoencephalitis) or IgA (neurotuberculosis, brain abscess, adrenoleukodystrophy) synthesis [9].

An artifact mimicking intrathecal Ig synthesis in quotient diagrams can be present due to blood contamination of the CSF [10]. Intrathecal IgM dominates, followed by IgA and IgG (QIgM > QIgA > QIgG) [9,10]. The occurrence of this Ig pattern in the presence of blood contamination is not to be mistaken as intrathecal inflammation. Since the smallest protein yields the highest CSF/serum quotients, it is less susceptible to concentration changes. A blood contamination of 5000 erythrocytes/µL CSF already results in a false-positive IgM synthesis in quotient diagrams in nearly every fifth patient, but not artificial IgA or -G synthesis [10]. It has not yet been described how or if FLC-k, as the smallest molecule (22.5 kDa), will complement the above-mentioned Ig pattern in case of blood contamination of the CSF. Only one recently published study has shown that blood spiking has no effect on absolute FLC-k concentrations in CSF [11]. The influence of blood spiking on the FLC-k representation in the quotient diagram, on the other hand, was not investigated [11].

The primary aim of this study was to determine whether intrathecal FLC-k synthesis occurs in patients with an intrathecal IgA and/or IgM synthesis in the absence of OCB, compared to a control group of patients with no signs of inflammation. Secondly, we evaluated the use of FLC-k as a potential marker to distinguish true Ig synthesis in quotient diagrams from the artifact induced by blood contamination.

## 2. Materials and Methods

The local institutional review board approved this study. This study was based solely on the use of coded surplus material. The use of coded surplus material without additional informed consent was based on the statement of the local ethics research committee (Vote III UV 39/03, University Medicine Greifswald, Greifswald, Germany). Between 2016 and 2018, we acquired samples for a prospective patient cohort as part of an already published study [7] comprised of paired CSF and serum samples from patients with isolated IgM and IgA synthesis, in the absence of IgG or patient samples fitting our control group. To increase the number of samples with isolated IgA and/or IgM synthesis, we additionally analysed paired CSF and serum samples, which had been collected between 2008 and 2016 and stored at −80 °C with isolated IgM or IgA synthesis. All samples were acquired from patients of the department of Neurology, University Medicine of Greifswald (Greifswald, Germany).

### 2.1. Laboratory Analysis

Laboratory analyses were performed in the interdisciplinary CSF laboratory of the University Medicine of Greifswald. Laboratory analyses were performed as described previously [7,12].

As a qualitative method to detect intrathecal IgG synthesis, the analysis of OCB was done by isoelectric focusing with a semiautomatic agarose electrophoresis system (Hydragel 9 CSF, Sebia Hydrasys 2Scan, Sebia GmbG, Fulda, Germany). As a quantitative method, the CSF serum quotients of FLC-k, IgG, IgA, and IgM were plotted against the albumin quotient in the already established quotient diagram [1,3].

FLC-k in the sera and CSF were measured by nephelometry with the N Latex FLC kappa kit (Siemens Healthcare Diagnostics Products GmbH, Marburg, Germany), using monoclonal antibodies on the BN Prospec analyser. CSF pre-dilution was set to 1:2, and serum pre-dilution was set to 1:100. The lower limit of quantification of the assay was 0.034 mg/L. Patients with highly elevated serum FLC-k values (FLC-k serum mean > 2 standard deviation) were excluded. The hyperbolic reference range, as well as the amount of intrathecally synthesized FLC-k, was calculated according to the formulas described by Reiber et al. [1,3]. The intrathecal fraction (*IF*) of Ig or FLC-k was calculated as described in Formula (1), with *Qlim* being the upper limit of the reference range:

*IF* = [1 − *Qlim*–FLC-k/(QFLC-k)] × 100
With QFLC-k as the CSF/serum quotient of FLC-k(1)

Patients were selected according to their CSF profile:

- Cohort I: isolated IgM or A synthesis (QIgM > Qlim-IgM, QIgA > Qlim-IgA) or combined IgA and IgM synthesis. No quantitative or qualitative IgG synthesis, and erythrocyte count <500/µL;
- Cohort II: blood contamination by traumatic lumbar puncture with QIgM > QlimIgM, and if present, QIgA > Qlim-IgA and QIgG > QLim-IgG but QIgM > QIgA > QIgG. Erythrocyte count was >500/µL and/or elevated ferritin, which was done in cases of suspected subarachnoid hemorrhage and always if the erythrocyte count was >100/µL. Ferritin is a very sensitive, while not a specific marker to detect intrathecal blood. The absence of elevated ferritin is a strong indicator of artificial blood contamination [13]. No patient sample had a quantitative or qualitative intrathecal IgG synthesis;
- Control group: no signs of inflammation in CSF profile; no pleocytosis or absence of intrathecal quantitative synthesis of IgM, IgA, IgG; and an absence of OCB in CSF.

To simulate artificial blood contamination, we used all patients of the control group that did not show an initial FLC-k synthesis (QFLC-k < *Qlim*–FLC-k). The mean amount of analysed CSF was estimated to be 2 mL. We used the individual serum albumin, IgG, IgA, IgM, and FLC-k values to simulate CSF contamination with 0.005 mL, 0.010 mL, 0.050 mL, and 0.100 mL of serum using the following formula:

ω = ((ω_1 × *m*_1) + (ω_2 × *m*_2))/(*m_*1 + *m*_2) (2)

where ω is the mass fraction and *m* represents the mass.

### 2.2. Statistical Analysis

RStudio (R version 3.5.1 2018-07-02) was used for statistical and graphical processing of the data. Statistical significance was assessed using Chi-square tests for nominal data. For a pairwise intergroup comparison, we performed the Kruskal–Wallis analysis of ranks test corrected for multiple tests; *p*-values ≤ 0.05 were regarded as statistically significant.

## 3. Results

A total of 296 patients were identified retrospectively, the samples analysed and assigned to one of three cohorts based on their CSF findings. Cohort I was comprised of 41 patients with either a combined intrathecal IgA and IgM synthesis or isolated intrathecal IgA or IgM synthesis. No patient sample presented signs of blood contamination as defined in cohort II. Cohort II was comprised of 16 patients with artificial IgM and/or IgA synthesis through blood contamination. The control group was comprised of 239 patients with neither intrathecal Ig synthesis nor isolated OCB in the CSF. Details of the CSF data are described in Table 1.

**Figure 1:**
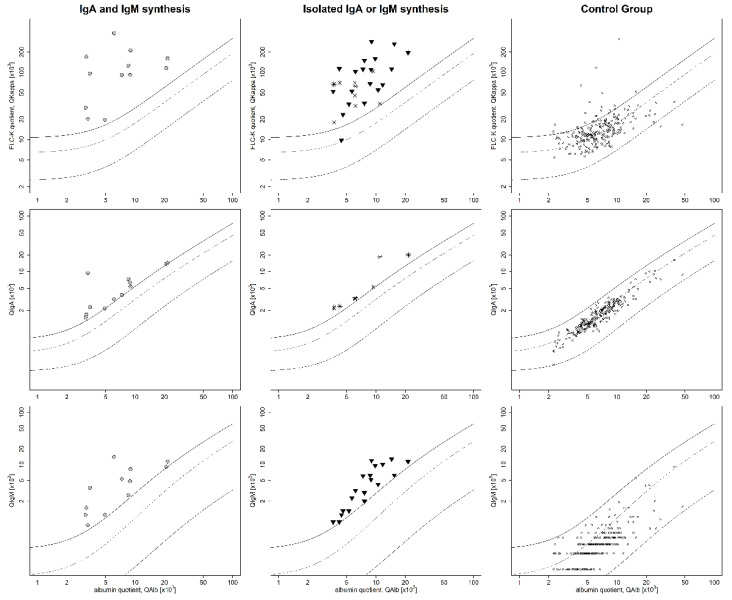
Data of cohort I and the control group in double logarithmic immunoglobulin (Ig)M, IgA, and free light chain kappa (FLC-k) quotient diagrams. The first column includes data from cohort I with combined IgA and IgM synthesis (circles). All FLC-k quotients are above *Qlim*–FLC-k. Column 2 contains the data of patient samples with an isolated IgA synthesis (asterisks) or isolated IgM synthesis (triangles). Of the FLC-k quotients, 98% are above *Qlim*–FLC-k. Column 3 displays the data of the control group (circles), in which 7.9% of the FLC-k quotients are above *Qlim*–FLC-k. The bold line shows *Qlim*, the dashed line shows the *Qmean*. The bottom dashed line shows the *Qlow*. *QLim*/*mean*/*low–*IgG/IgA/IgM/FLC-k = upper/mean/lower limit of the hyperbolic reference range; IF = intrathecal fraction.

Figure 1 shows the FLC-k and IgA/IgM quotients in corresponding quotient diagrams of cohort I and the control group. In cohort I, all patient samples with intrathecal IgA and IgM synthesis (Figure 1, column 1) presented an FLC-k quotient above *Qlim*–FLC-k. Furthermore, all patient samples with an isolated intrathecal IgA synthesis (Figure 1, column 2, indicated by asterisks) had a FLC-k quotient > Qlim-FLC-k. One out of 19 patient samples (5.2%) with isolated IgM synthesis (Figure 1, column 2, indicated by triangles) had no evidence of intrathecal FLC-k synthesis (QFLC-k < Qlim-FLC-k).

In our cohorts, FLC-k analysis confirms “true” Ig synthesis, with a high sensitivity of 98%. Even though no patient in the control cohort had an intrathecal IgA or IgM synthesis, according to the quotient diagram, and no OCB, 19 patients (7.9%) had an FLC-k quotient > *Qlim*–FLC-k, suggesting intrathecal FLC-k synthesis (Figure 1, column 3).

**Figure 2:**
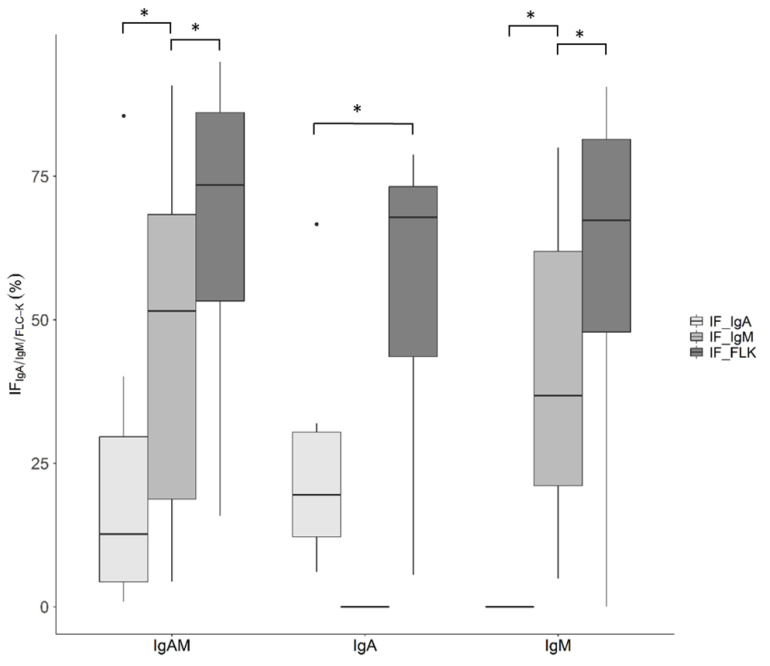
Boxplot of the IF IgA/IgM/FLC-k of cohort 1. Cohort I is divided into isolated IgA and combined IgA and IgM synthesis in the quotient diagram. In both cohorts, differences between IF IgA, IF IgM, and IF FLC-k were significant. * *p*-value < 0.05 (from left to right: *p =* 0.0001, *p* = 0.08, *p* = 0.01, *p* < 0.001, and *p* = 0.006). IF = intrathecal fraction.

Figure 2 shows that the true intrathecal synthesis of IgA and IgM is accompanied by a significantly higher IF of FLC-k compared with the IF of IgA or IgM.

**Figure 3:**
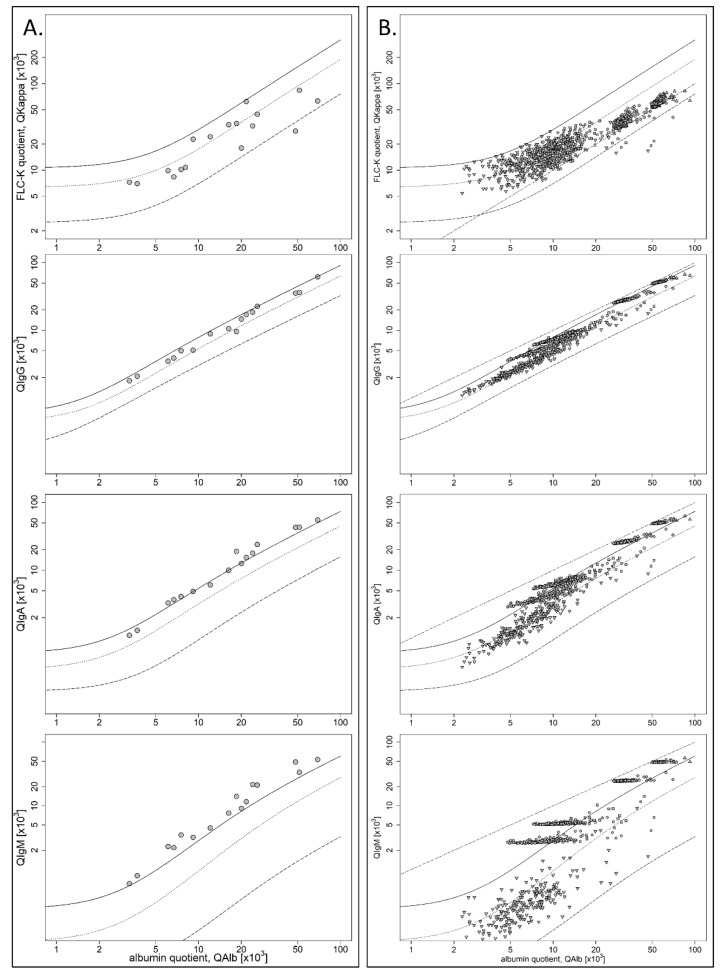
(**A**) Data of cohort II in double logarithmic IgG, IgA, IgM, and FLC-k quotient diagrams. All patients had a QFLC-k below the corresponding *Qlim*–FLC-k. (**B**) Data of the control group with a QFLC-k < *Qlim*–FLC-k (∇) and simulated blood contamination with 0.005 mL (○), 0.010 mL (□), 0.050 mL (◊), and 0.100 mL (∆) of serum in double-logarithmic IgG, IgA, IgM, and FLC-k quotient diagrams. QFLC-k = free light chain kappa quotient, *Qlim*–FLC-k = upper limit of the hyperbolic reference range.

Figure 3A shows the FLC-k, IgG, IgA, and IgM quotient diagrams for cohort II. All patient samples of this cohort presented an FLC-k quotient < *Qlim*–FLC-k, meaning that FLC-k quotients are not elevated in cases of artificial Ig synthesis through blood contamination. One patient sample showed a QFLC-k below the lower limit of the reference range (*Qlow*–FLC-k).

Figure 3B shows the FLC-k, IgG, IgA, and IgM quotient diagrams for the control group, excluding the patient samples with an initial QFLC-k > Qlim-FLC-k after simulating blood contaminations of varying extent (*n* = 220). The dot–dashed line represents the bisecting line (*y* = *x*), which is progressively approached by the patient samples with increasing simulated blood contaminations. Table 2 shows the number of patient samples that showed a QIgG/IgA/IgM/FLC-k > *Qlim*–IgG/-IgA/-IgM/-FLC-k after simulating blood contamination of 2 mL CSF and with 0.005 mL, 0.010 mL, 0.050 mL, and 0.100 mL of serum. With 0.100 mL of serum contamination in the CSF, 99% of all patient samples would present artificial IgM or IgA synthesis, and 89% would present an artificial IgG synthesis, compared to no patient sample with artificial FLC-k synthesis.

**Figure 4:**
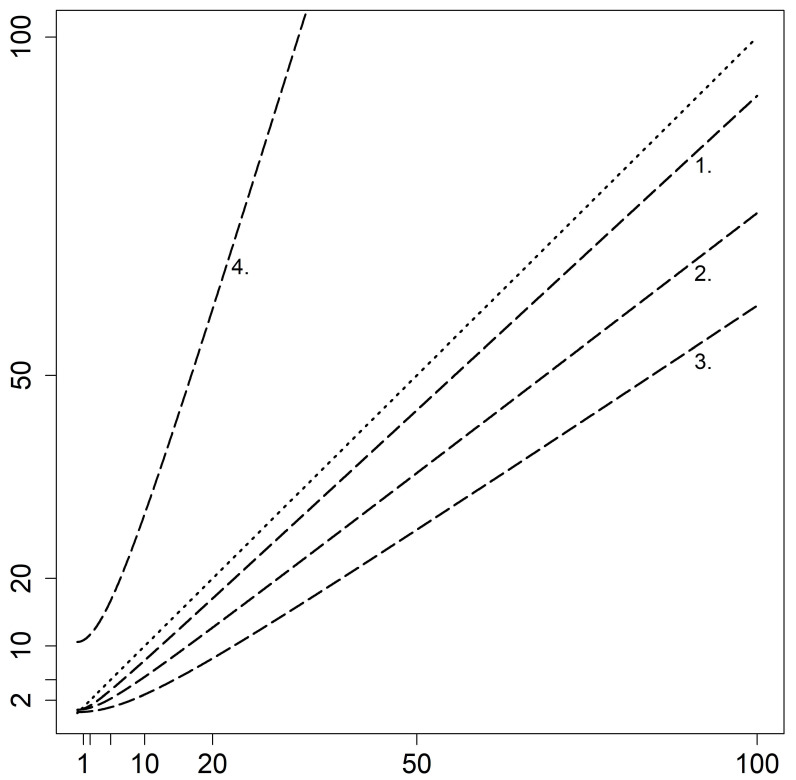
Display of the upper reference limit (*Qlim*) for IgG, IgA, IgM, and FLC-k in relation to the linear function. The steep increase of the *Qlim* (FLC-k) graph is contrasted by the lower increase of the graph for the *Qlim* (IgG, IgA, IgM), which results in *Qlim*–IgG (1.), *Qlim*–IgA(2.), *Qlim*–IgM (3.) and *Qlim*–FLC-k (4.). *Qlim*–IgG/-IgA/-IgM/-FLC-k = upper limit of the hyperbolic reference range.

Figure 4 shows the functions of *Qlim* for IgG, IgA, IgM, and FLC-k, with increasing *Qalb*. Immunoglobulin G (IgG), IgA, and IgM indicate a similar progression, as already described by Reiber et al. [1]. The *Qlim*–FLC-k, though, is situated above the bisecting line, and shows a different progression than the immunoglobulins, resulting in a much higher slope.

## 4. Discussion

In several studies, the determination of FLC-k is described as a similar sensitive method to the detection of OCB, with isoelectric focusing with lesser specificity [7,14,15]. As OCB analysis represents exclusively an intrathecal IgG synthesis, the question arises if FLC-k can also represent intrathecal IgM and/or IgA synthesis, as pathophysiological considerations suggest. This theoretical concept could be confirmed in this study: An increased FLC-k quotient above the hyperbolic reference range can be found in 98% of our studied samples with intrathecal IgM or IgA synthesis, while a quantitative and qualitative proof of IgG is absent.

### 4.1. FLC-k as a CSF Biomarker for Inflammation

It has already been shown that FLC-k is a potential biomarker for intrathecal inflammation in multiple sclerosis (MS) and clinical isolated syndrome (CIS), and that it can help to discriminate between inflammatory and non-inflammatory CNS diseases [12,15,16]. Furthermore, an elevated FLC-k index can be found in neuroborreliosis [17] and HIV [18]. Our data suggest that FLC-k is a non-specific biomarker of intrathecal inflammation, representing intrathecal IgG but also an intrathecal IgA and IgM synthesis. Therefore, FLC-k can be used as a marker for not only IgG dominant inflammatory diseases like MS, but also for IgM- (e.g., neuroborreliosis) or IgA-dominant diseases (e.g., neurotuberculosis). This concept is supported by biomarker studies of haematological diseases, e.g., IgM paraproteinaemias, showing that a FLC-k detection in sera can be a product of pathological IgM secretion [19].

In recent works, FLC-k has been emphasised to have a higher sensitivity to the quantitative analysis of IgG in quotient diagrams, and a comparable sensitivity to detect intrathecal IgG synthesis by OCB analysis [7,14,15,16]. While an up to 200% higher amount of IgG might be needed to indicate intrathecal synthesis in quotient diagrams in physiologically low IgG levels, only 0.5% of total synthesised IgG is needed for OCB detection in CSF [20]. Even though patients with physiologically normal FLC-k values have to synthesise up to 136% of their original FLC-k concentrations to reach *Qlim*–FLC-k [3], the still-comparable sensitivity to OCB analysis in IgG-dominant neurological diseases has been proven [7,14,15]. Whether the sensitivity of FLC-k analysis is similar in cases of intrathecal IgA or IgM synthesis is not known, mainly because a sufficiently sensitive method comparable to OCB is lacking [21,22]. The sensitivity and specificity of measuring IgM by isoelectric focusing is compromised, since the pentamers of an IgM clone have to be broken down into monomers before analysis, and then associate again in the gel in random combinations of arbitrarily different clones. The intensity of such bands correlates with the amount of IgM in the CSF, and is not corrected for blood CSF barrier dysfunction. Therefore, the method is prone to false negative and false positive findings [22]. Evidence for a higher sensitivity of the FLC-k Reibergram to detect an IgM- or IgA-dominant intrathecal inflammation comes from a significantly higher IF of FLC-k compared to the IF of IgM or IgA (see Figure 2). This indicates that already, low levels of intrathecal synthesised IgA or IgM are accompanied by a QFLC-k above the *Qlim–*FLC-k, and a high level of IF FLC-k as a result.

The majority of patients in the control cohort have FLC-k values below *Qlim*–FLC-k. However, 6.5 % of the patients show intrathecal FLC-k synthesis. This FLC-k positivity of unknown origin could be due to the already-described low sensitivity of the quotient diagram for IgM or IgA. Moreover, the IgM quotient is prone to be influenced by measurement inaccuracy, due to the low CSF concentration [20] by which an intrathecal IgA or IgM synthesis could have simply been missed. Another reason for isolated intrathecal FLC-k synthesis could be a putative intrathecal IgE [23] or IgD [24] synthesis, which is not part of the recommended diagnostic procedure [25] and hence was not part of the conducted routine CSF diagnostic.

In summary, the FLC-k quotient, accompanied by the established hyperbolic reference range, can not only be used to detect intrathecal inflammation, comprised of intrathecal IgG synthesis with a similar sensitivity to OCB analysis, but can also be an indicator for an inflammatory process with an intrathecal IgA or IgM synthesis.

### 4.2. FLC-k in the Context of Artificial Blood Contamination

Our data (see Figure 3A) show that all patient samples with blood contamination, QIgM > QIgA, and no IgG synthesis have QFLC-k values below the upper reference limit (QFLC-k < *Qlim*–FLC-k). This empirical data is supported by our simulated data (see Figure 3B). With an increase in simulated blood contamination, the typical pattern of artificial synthesis for IgG/IgA/IgM develops, which was also recently demonstrated by Schwenkenbecher et al. [10]. The QFLC-k, however, remains below the *Qlim*–FLC-k. These results can be explained as follows.

A blood contamination of CSF samples bypasses the molecular size-related selectivity of the blood–CSF barrier. FLC-k and Ig CSF/serum quotients stop following the hyperbolic reference range, but increasingly follow a linear function (bisecting line; *y* = *x*, see Figure 3B and Figure 4), as already described by the “leakage” metaphor by Reiber et al. [26]. The *Qlim*–IgG/-IgA/-IgM is situated below (and the QFLC-k above) the linear function (see Figure 4). In case of a blood contamination, quotients approach the bisecting line, which results in an artificial synthesis of IgG/IgA/IgM, but not FLC-k. With increasing blood contamination, the QFLC-k values also approach the bisecting line. This results in the distancing of the QFLC-k values from the *Qlim*–FLC-k (as seen in Figure 3B).

This also explains the one patient sample with the QFLC-k below the *Qlow*–FLC-k. As a consequence of the blood contamination, the quotients follow the linear function and drop out of the hyperbolic reference range.

FLC-k quotients above the upper reference limit in quotient diagrams (QFLC-k > Qlim-FLC-k) reflect intrathecal inflammation with high sensitivity [3]. In addition, test results that show no intrathecal FLC-k synthesis (QFLC-k < *Qlim*–FLC-k) while the quotient diagrams for IgM, IgA, and IgG result in apparent intrathecal Ig synthesis, are results of blood contamination. Adding FLC-k evaluation to CSF analysis can thus help to distinguish the effects of artificial blood contamination from intrathecal Ig synthesis in patient samples with QIgM > QIgA (>QIgG).

One major limitation of this study is the small sample size for the constellation of an isolated IgA or IgM synthesis, which is due to the rare appearance of this protein pattern.

## 5. Conclusions

We could show that FLC-k synthesis can be found in patients with intrathecal IgA or IgM synthesis, with a sensitivity of 98%. However, in patients with artificial intrathecal Ig synthesis due to blood contamination, FLC-k synthesis is lacking. Thus, an additional determination of FLC-k in quotient diagrams helps to distinguish an inflammatory CNS process from an artificial Ig synthesis due to blood contamination of the CSF.

## Figures and Tables

**Table 1:** Baseline characteristics and cerebrospinal fluid results.

	Patient Cohort I (Intrathecal Synthesis)	Patient Cohort II (Blood Contamination)	Control Group	*p*-Value
*n*	41	16	239	
Age	40 (28; 59)	58 (45; 77)	52 (37; 68)	
Female (%)	44	62	52	
CC (/µL)	23 (3; 48)	16 (6; 52)	1 (1; 1)	<0.0001
EC (/µL)	9 (1; 60)	4466 (2233; 28750)	1 (0;4)	<0.0001
Ferritin ng/mL	-	11.3 (6.1; 26.8)	-	
QAlb	7.3 (4.6; 9.3)	17.4 (7.3; 24.5)	6.1 (4.7; 8.6)	0.02
QIgM	2.6 (1.1; 6.3)	8.4 (2.9; 21.3)	0.3 (0.2; 0.4)	<0.0001
QIgA	3.1 (2.2; 5.3)	11.3 (4.0; 20.2)	1.5 (1.1; 2.4)	<0.0001
QIgG	3.5 (2.7; 5.0)	10.1 (4.7; 19.6)	3.1 (2.3; 4.2)	<0.0001
IF IgM (%)	21 (4; 60)	24 (18; 45)	0 (0; 0)	<0.0001
IF IgA (%)	4 (0; 17)	9 (0; 13)	0 (0; 0)	<0.0001
IF IgG (%)	0 (0; 0)	0 (0; 0)	0 (0; 0)	<0.0001
FLC-k serum mg/L	15.30 (11.40; 18.90)	16.80 (14.05; 23.07)	12.40 (9.45; 16.10)	0.001
FLC-k CSF mg/L	1.29 (0.65; 1.81)	0.40 (0.17; 0.80)	0.20 (0.11; 0.31)	<0.0001
QFLC-k	91.4 (45.1; 125.8)	26.4 (10.12; 37.1)	12.7 (10.2; 17.5)	<0.0001
IF FLC-k (%)	68 (47; 79)	0 (0; 0)	0 (0; 0)	<0.0001
QFLC-k > *Qlim–*FLC-k (*n* (%))	40 (97.6)	0 (0)	19 (6.5)	

Continuous data are expressed as median (first; third quartiles); nominal data are given as percentages. EC = erythrocyte count, CC = cell count (leukocytes), QIgG/A/M = immunglobulin G/A/M quotient, QFLC-k = free light chain kappa quotient, *Qlim*–IgG/-IgA/-IgM/-FLC-k = upper limit of the hyperbolic reference range, IF IgG/IgA/IgM/FLC-k = intrathecal fraction.

**Table 2:** Number of patient data developing a QIgG/IgA/IgM/FLC-k > *Qlim*–IgG/IgA/IgM/FLC-k in the quotient diagram after simulating blood contamination of 2 mL cerebrospinal fluid (CSF) with the given volume of serum.

Added Volume of Serum	QIgG > *Qlim*–IgG; *n* (%)	QIgA > *Qlim*–IgA; *n* (%)	QIgM > *Qlim*–IgM; *n* (%)	QFLC-k > *Qlim*–FLC-k
0.005 mL	14 (6.3)	66 (30)	142 (64.5)	0
0.010 mL	28 (12.7)	127 (57.7)	189 (85.9)	0
0.050 mL	138 (62.7)	213 (96.8)	215 (97.7)	0
0.100 mL	195 (88.6)	218 (99)	218 (99)	0

QIgG/A/M = Immunglobulin G/A/M quotient, QFLC-k = free light chain kappa quotient, *Qlim*–IgG/-IgA/-IgM/-FLC-k = upper limit of the hyperbolic reference range.

## Data Availability

The data presented in this study are available on request from the corresponding author. The data are not publicly available.
